# Gestational Weight Gain and Overweight in Children Aged 3–6 Years

**DOI:** 10.2188/jea.JE20140149

**Published:** 2015-08-05

**Authors:** Lianhong Guo, Jufen Liu, Rongwei Ye, Jianmeng Liu, Zhixiong Zhuang, Aiguo Ren

**Affiliations:** 1 Department of Health Toxicology, School of Public Health, Central South University, Changsha, Hunan, China; 2 Institute of Reproductive and Child Health/Ministry of Health Key Laboratory of Reproductive Health, School of Public Health, Peking University, Beijing, China; 3 College of Science, Northwest A&F University, Yangling, Shaanxi, China; 4 Center for Disease Control and Prevention, Shenzhen, Guangdong, China

**Keywords:** cohort study, gestational weight gain, childhood overweight, birth weight, maternal pre-pregnancy BMI

## Abstract

**Objective:**

To determine whether gestational weight gain (GWG) was associated with increased odds of childhood overweight after accounting for pre-pregnancy BMI.

**Methods:**

In a prospective cohort study based on a premarital and perinatal health care system in China, data of 100 612 mother-child pairs were obtained. The main exposure was GWG as both a continuous and categorical variable. The outcome measure was overweight, defined by age- and sex-specific cutoff values for body mass index (BMI) in children aged 3–6 years.

**Results:**

A 1-kg increase in maternal GWG was associated with an increase of 0.009 (95% confidence interval [CI]: 0.007–0.010, *P* < 0.001) in children’s mean BMI; in the subgroup of pre-pregnancy overweight/obese mothers, the increase in children’s BMI was 0.028 (95% CI, 0.017–0.039, *P* < 0.001). Excessive GWG played an important role in childhood overweight when adequate GWG was used as the reference, with an odds ratio (OR) of 1.21 (95% CI, 1.12–1.29). The risk was highest (OR 2.22; 95% CI, 1.79–2.76) in the children of mothers who were overweight/obese before pregnancy and gained excessive weight during pregnancy.

**Conclusions:**

Greater maternal GWG was associated with greater offspring BMI, and the risk of overweight was doubled in children whose mothers were overweight/obese before pregnancy and gained excessive weight during pregnancy. As a result, maintenance of appropriate weight gain during pregnancy and prophylaxis of maternal overweight/obesity before pregnancy should be a strategy for preventing childhood overweight/obesity.

## INTRODUCTION

Childhood obesity has become a serious public health problem and has attracted much attention across all geographic areas, ethnic backgrounds, and cultures in the last three decades.^1^^–^^7^ Obese children are more likely to become obese adults than non-obese children.^8^^,^^9^ Childhood obesity is linked to both short-term and long-term health consequences, including cardiovascular events, coronary heart disease, hypertension, some types of cancer, dyslipidemia, diabetes mellitus, gallbladder disease, respiratory disease, gout, and arthritis.^10^^–^^14^ Genetic, environmental, and lifestyle factors account for most of the overweight prevalence in children; these include parental obesity, gestational age, birth order, socioeconomic status, residence in a single-parent household, watching large amounts of television, and lack of physical activity.^15^^–^^22^

In China, rapid economic growth, lack of physical activity, and the replacement of a traditional Chinese diet with a “Western diet” have led to a serious obesity problem. A systematic review showed that there is an increasing trend in the prevalence of obesity in preschool children, from 0.7% in 1986–1990 to 6.8% in 2006–2010.^3^ In the meantime, China Health and Nutrition Survey data have indicated that weight status in adults is also troublesome. There were increases of 1.2 kg/m^2^ in BMI, 67.0% in the prevalence of overweight, and 168.0% in the prevalence of obesity between 1993 and 2009.^23^ Therefore, we need to take effective actions to curb this trend.

Gestation is a crucial period of time during which both mothers and children grow, develop, and undergo physiological changes. Many studies have shown that childhood obesity has roots in overnutrition during fetal development.^24^^,^^25^ Several studies have suggested that there is an association between gestational weight gain (GWG) and downstream weight status in childhood and throughout adulthood.^6^^,^^26^^–^^29^ However, the existing evidence does not allow inferences to be drawn; sample sizes and age groups vary markedly among studies, which may influence the precision of the estimates. Moreover, different reference groups, Institute of Medicine guidelines on maternal GWG, and cutoff values have been used in different studies. Furthermore, it is not clear whether the association between maternal GWG and childhood overweight is linear or non-linear. We hypothesized that maternal GWG would have a linear relationship with offspring’s risk of overweight. Indeed, Oken found that gestational weight gain was linearly associated with adolescent adiposity in the Growing Up Today Study.^30^ However, other studies have reported a U- or J-shaped association where greater overweight risks were found in the lowest and highest maternal gains in U-shaped association, while normal maternal gain had less risk of offspring overweight than inadequate maternal gain, and excessive maternal gain has the highest risk of offspring overweight in J-shaped association.^31^^,^^32^

We used a prospective cohort consisting of 100 612 mother-child pairs to examine the association between maternal GWG and overweight in children aged 3–6 years from 16 counties or cities in China, which has been the second largest population of overweight people after the United States according to the latest statistics released by the CASS.

## METHODS

### Study population and data sources

We used data from a population-based premarital and perinatal health care system that was universally implemented in 16 counties or cities in two southern China provinces (Zhejiang and Jiangsu provinces). Women who entered the premarital health assessment program between December 1992 and February 1996 were enrolled before they got married, and they were followed until 42 days postpartum. Information on demographic and relevant obstetric and pregnancy outcomes, such as maternal age, education, occupation, gravidity, and weeks of gestation were collected at the time of entry, subsequent prenatal visits, or delivery. When a child was born, information on sex, birth length, and birth weight were collected. These children were born between October 1993 and December 1996. We followed the children from birth until between March and July 2000. The mean (standard deviation [SD]) follow-up between the date of birth and the date of child follow-up examination was 4.4 (3.8) years. Child’s age (in months) was calculated by the date of follow-up examination minus the date of birth. At the follow-up examination, we measured each child’s weight (kg) and height (cm), with the subjects wearing light indoor clothing without shoes or hats. Calibration of all instruments and quality control of the measurements were performed by local experts. All work was completed by trained local health care workers using a standard protocol.

The initial sample included 114 161 mother-child pairs. After exclusion of 13 549 pairs, as shown in Figure, we obtained a final cohort with 100 612 (88.1%) mother-child pairs. The Peking University Institutional Review Board approved this study.

**Figure.**
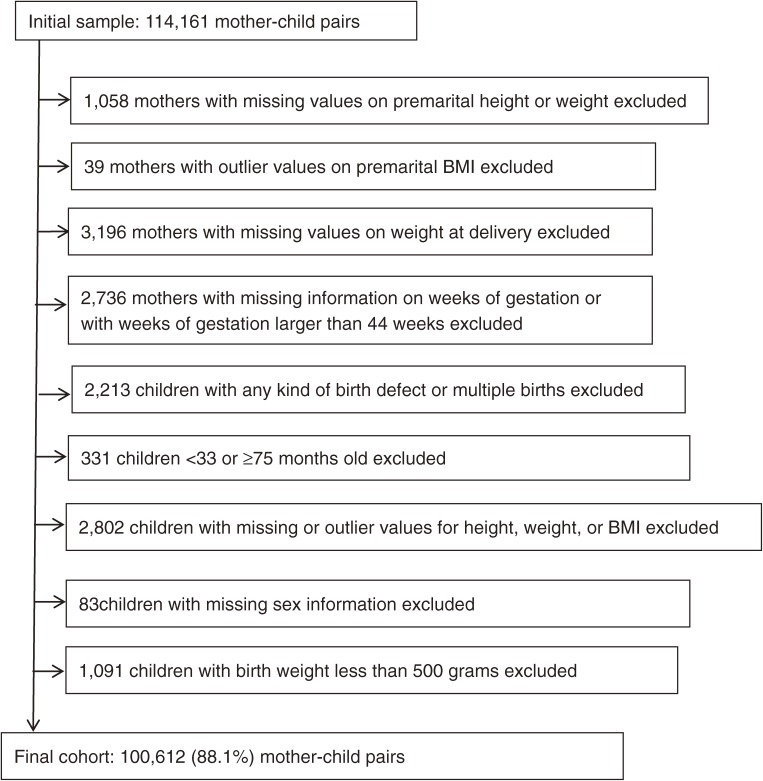
Flow chart of study selection.

### Explanatory variables

The exposure to be examined was GWG as both a continuous and categorical variable. Total GWG was calculated as the difference between the weight at delivery and pre-pregnancy weight (weight at the premarital medical examination). Weight at the premarital medical examination and delivery were measured by trained health professionals. GWG was classified as inadequate, adequate, or excessive according to the IOM guidelines on GWG, which were revised in 2009 and are suitable for Chinese people.^33^^,^^34^ We combined overweight and obese mothers because the number of obese mothers was very small. Pre-pregnancy BMI (kg/m^2^) was calculated with maternal weight and height values obtained from the premarital examination, and we defined women as underweight (BMI <18.5), normal weight (BMI 18.5–24.9), and overweight/obese (BMI ≥25.0) according to the current World Health Organization BMI cut-off points.^35^^,^^36^ Childhood overweight was defined according to international criteria on age- and sex-specific cutoff values for BMI.^37^ Specifically, cutoffs of 17.89, 17.69, 17.55, 17.47, 17.42, 17.45, and 17.55 were adopted for boys at ages 3 (33–38 months), 3.5 (39–44 months), 4 (45–50 months), 4.5 (51–56 months), 5 (57–62 months), 5.5 (63–68 months), and 6 (69–74 months) years, respectively. Respective cutoffs were 17.56, 17.40, 17.28, 17.19, 17.15, 17.20, and 17.34 for girls at ages 3, 3.5, 4, 4.5, 5, 5.5, and 6 years.

### Statistical analyses

We used chi-square test to compare characteristics of the mothers and children aged 3–6 according to maternal GWG and the association between covariates and children’s overweight. We used linear regression to examine the association of GWG (as a continuous variable) with children’s BMI and binary logistic regression to examine the relationship of GWG (as a categorical variable) with the risk of childhood overweight. Included covariates were child sex, age, and birth weight, as well as maternal age, education, occupation, gravidity, weeks of gestation, and year of registry. Odds ratios (ORs) and their corresponding 95% confidence intervals (CIs) for childhood overweight were calculated from logistic regression models, with adjustment for the potential confounders. We performed all analyses using SPSS version 19.0 (SPSS Inc., Chicago, IL, USA). A two-sided *P*-value <0.05 was considered statistically significant.

## RESULTS

The anthropometric data for mothers and children and the proportion of subjects in each GWG category are shown in Table 1. Upon entering pregnancy, 81.2% of the 100 612 mothers were of normal weight, 16.5% were underweight, and 2.3% were overweight/obese. 63.7% mothers had finished junior high school education and 11.5% had finished high school or college, more than half (53.4%) were farmers, 54.9% were primigravidas, and 95.4% were nulliparous. About 51.6% of the children were boys, 7.2% were born preterm, and 1.9% were born at a low birth weight. Mean (SD) maternal age was 23.9 (2.1) years, mean (SD) pre-pregnancy BMI was 20.3 (2.0) kg/m^2^, and mean (SD) total GWG was 11.7 (5.0) kg. Inadequate GWG was present in 50.6% of the mothers, 34.9% showed adequate GWG, and 14.5% showed excessive GWG according to the IOM criteria. About 54.3% of farmer mothers gained inadequate weight during pregnancy, which was significantly higher than mothers who were factory workers or worked other jobs. The proportion of mothers who gained inadequate weight was also higher in the elementary or no education group than in the junior high school group and high school or college group. Further, the proportion of mothers who gained excessive weight was higher in the higher pre-pregnancy BMI group than in normal and underweight pre-pregnancy groups. The proportion of mothers with excessive GWG was highest in the child age group 33–44 months and lowest in child age group 69–74 months.

**Table 1.** Characteristics of the mothers and children aged 3–6 years according to maternal GWG in 16 Chinese counties or cities

Characteristic	*n* (%) or mean (SD)	GWG category (%)/mean (SD)			*P*-value^a^

		Inadequate	Adequate	Excessive	
Number of subjects	100 612 (100)	50.6	34.9	14.5	
Maternal age, years	23.9 (2.1)	23.9 (2.0)	24.0 (2.1)	24.0 (2.1)	0.051
Gestational weight gain, kg	11.7 (5.0)	7.9 (2.6)	13.8 (1.7)	19.7 (3.9)	<0.001
Pre-pregnancy BMI, kg/m^2^	20.3 (2.0)	20.6 (2.0)	20.0 (2.0)	20.1 (2.2)	<0.001
Pre-pregnancy BMI, kg/m^2^					<0.001
<18.5	16 604 (16.5)	43.2	43.3	13.5	
18.5–24.9	81 725 (81.2)	52.4	33.2	14.4	
≥25.0	2292 (2.3)	38.0	33.6	28.4	
Highest education					<0.001
High school or college	11 518 (11.5)	40.4	39.8	19.8	
Junior high school	63 898 (63.7)	50.4	35.0	14.6	
Elementary or none	24 952 (24.8)	55.7	32.2	12.1	
Occupation					<0.001
Farmer	53 645 (53.4)	54.3	33.1	12.6	
Factory worker	41 509 (41.3)	47.5	36.4	16.0	
Other	5336 (5.3)	37.2	40.6	22.2	
Gravidity					<0.001
First pregnancy	55 201 (54.9)	53.7	33.0	13.3	
Second or later pregnancy	45 367 (45.1)	46.8	37.1	16.1	
Parity					<0.001
First delivery	95 976 (95.4)	50.5	34.9	14.6	
Second or later delivery	4601 (4.6)	53.6	33.7	12.7	
Gestation, weeks					<0.001
<37	7131 (7.2)	62.8	27.3	9.9	
≥37	92 570 (92.8)	49.7	35.4	14.9	
Child birth weight, grams					<0.001
<2500	1922 (1.9)	73.9	21.1	5.0	
≥2500	98 690 (98.1)	50.1	35.1	14.8	
Child sex					<0.001
Female	48 728 (48.4)	51.4	34.7	13.9	
Male	51 884 (51.6)	49.8	35.1	15.1	
Child age groups, months					<0.001
33–44	16 319 (16.2)	43.7	38.2	18.1	
45–56	48 605 (48.3)	47.6	36.7	15.7	
57–68	34 901 (34.7)	57.4	31.1	11.4	
69–74	787 (0.8)	71.8	21.1	7.1	

BMI, body mass index; GWG, gestational weight gain; SD, standard deviation.

^a^The *P*-value of Pearson’s chi-squared test.

As shown in Table 2, the prevalence of overweight was higher in children of pre-pregnancy overweight/obese mothers than in those of normal-weight and underweight mothers. Mothers with excessive GWG had a higher rate of overweight children than mothers with adequate or inadequate GWG. The prevalence of overweight was higher in children of second or later pregnancy mothers than in those of first pregnancy mothers. Girls had a higher proportion of overweight than boys. Prevalence of overweight was greater among children with normal birth than children with inadequate birth weight. The prevalence of overweight among children was also higher in the younger child age group than in older groups, and it increased with year of registry.

**Table 2.** Association between covariates and children’s overweight in 16 counties or cities in China

Characteristic	Children *n* (%)		*P*-value^a^

	Normal	Overweight	
Maternal age, years			0.372
15–20	7387 (92.3)	605 (7.7)	
21–24	71 121 (93.0)	5483 (7.0)	
25–30	13 684 (92.9)	1049 (7.1)	
≥31	1236 (92.8)	97 (7.2)	
Pre-pregnancy BMI, kg/m^2^			<0.001
<18.5	15 700 (94.6)	912 (5.4)	
18.5–24.9	75 755 (92.7)	6054 (7.3)	
≥25.0	2017 (88.0)	275 (12.0)	
GWG			<0.001
Inadequate	47 634 (93.6)	3257 (6.4)	
Adequate	32 519 (92.7)	2574 (7.3)	
Excessive	13 319 (91.1)	1309 (8.9)	
Gravidity			<0.001
First pregnancy	51 424 (93.2)	3777 (6.8)	
Second or later pregnancy	42 005 (92.6)	3362 (7.4)	
Parity			0.319
First delivery	89 152 (92.9)	6824 (7.1)	
Second or later delivery	4286 (93.2)	315 (6.8)	
Gestation, weeks			0.653
<37	6642 (93.1)	495 (6.9)	
≥37	85 993 (92.8)	6577 (7.1)	
Child birth weight, grams			0.002
<2500	1823 (94.8)	99 (5.2)	
≥2500	91 649 (92.9)	7041 (7.1)	
Child sex			0.004
Female	45 113 (92.6)	3615 (7.4)	
Male	48 359 (93.2)	3525 (6.8)	
Child age groups, months			<0.001
33–44	14 901 (91.3)	1418 (8.7)	
45–56	44 900 (92.4)	3705 (7.6)	
57–68	32 912 (94.3)	1989 (5.7)	
69–74	759 (96.4)	28 (3.6)	
Year of registry			<0.001
1993	62 (96.9)	2 (3.1)	
1994	23 191 (94.7)	1290 (5.3)	
1995	44 471 (93.2)	3259 (6.8)	
1996	25 748 (90.9)	2589 (9.1)	

BMI, body mass index; GWG, gestational weight gain.

^a^The *P*-value of Pearson’s chi-squared test.

In order to determine whether childhood obesity was linearly or non-linearly related to GWG, we divided GWG into 6 groups (<5.0, 5.0–9.9, 10.0–14.9, 15.0–19.9, 20.0–24.9, and ≥25 kg) and calculated the children’s mean BMI of each group. We found children’s mean BMI (15.41, 15.49, 15.58, 15.65, 15.73, and 15.83) increased in the 6 GWG groups, from least to most weight gained groups. In the linear regression model without adjustment, GWG was associated with an increase in children’s mean BMI, both in all mothers and in the three maternal pre-pregnancy BMI subgroups (Table 3). These associations remained after adjustment for child sex, age, and birth weight, as well as maternal age, education, occupation, gravidity, weeks of gestation, and year of registry. For example, a 1-kg increase in the GWG of all mothers resulted in a mean increase of 0.009 BMI units in their offspring. Moreover, in the subgroup of overweight/obese mothers, 1 kg of GWG accounted for an average increase in children’s BMI of 0.028.

**Table 3.** Linear regression estimates for the association of maternal GWG with children’s BMI at age 3–6 years according to the pre-pregnancy BMI category

Pre-pregnancy BMI groups	Unadjusted coefficient (95% CI)	Adjusted^a^ coefficient (95% CI)
All mothers	0.009 (0.007, 0.010)	0.017 (0.015, 0.018)
Underweight mothers	0.014 (0.010, 0.018)	0.022 (0.018, 0.026)
Normal weight mothers	0.011 (0.009, 0.013)	0.019 (0.017, 0.021)
Overweight/Obese mothers	0.028 (0.017, 0.039)	0.034 (0.023, 0.045)

BMI, body mass index; CI, confidence interval; GWG, gestational weight gain.

^a^Adjusted for child sex, age, child birth weight, maternal age, education, occupation, gravidity, weeks of gestation, and year of registry.

Table 4 shows the relative risks of childhood overweight stratified by maternal pre-pregnancy BMI and GWG. After adjustment for all confounding factors mentioned above, the odds of childhood overweight were significantly higher in children born to mothers with pre-pregnancy overweight/obesity than in those born to mothers with normal weight. Excessive GWG was associated with an overall increase of 21% in the odds of childhood overweight compared with adequate GWG. In addition, we analyzed the risk of childhood overweight according to nine joint groups of maternal pre-pregnancy BMI and GWG. Children born to mothers with both pre-pregnancy normal weight and adequate GWG were used as the reference group. Excessive GWG was associated with an increased risk of offspring overweight in normal weight and overweight/obese mothers. Meanwhile, adequate GWG was also associated with an increased risk of childhood overweight in overweight/obese mothers. These results suggest that adequate GWG may also be associated with increased risk of childhood overweight in the overweight/obese group. Of further note, children of mothers who were overweight/obese before pregnancy and displayed excessive GWG were 122% more likely to be overweight.

**Table 4.** Binary logistic regression estimates for the association of maternal GWG with children’s overweight at age 3–6 years stratified by maternal pre-pregnancy BMI^a^

Pre-pregnancy BMI	GWG categories, OR (95% CI)			Total

	Inadequate	Adequate	Excessive	
BMI < 18.5	0.62 (0.55, 0.70)	0.69 (0.61, 0.76)	0.93 (0.79, 1.10)	0.71 (0.66, 0.77)
BMI 18.5–24.9	0.91 (0.85, 0.96)	1.00	1.13 (1.04, 1.22)	1.00
BMI ≥ 25.0	1.18 (0.92, 1.50)	1.78 (1.43, 2.22)	2.22 (1.79, 2.76)	1.73 (1.52, 1.97)
Total	0.91 (0.86, 0.97)	1.00	1.21 (1.12, 1.29)	

BMI, body mass index; CI, confidence interval; GWG, gestational weight gain; OR, odds ratio.

^a^Adjusted for child sex, age, child’s birth weight, maternal age, education, occupation, gravidity, weeks of gestation, and year of registry.

## DISCUSSION

In this large prospective cohort study, we found that maternal GWG was linearly associated with the BMI of children aged 3–6 years. The odds of childhood overweight were 21% greater for the children of mothers who gained more than the weight gain recommendations than for children of mothers who met the weight gain recommendations.

Our results are consistent with several studies supporting a positive association between GWG and childhood obesity. A meta-analysis by Tie showed that, after eliminating the effect of publication bias by trim and fill analyses, the odds of excessive GWG and childhood overweight still remained statistically significant (OR 1.21; 95% CI, 1.05–1.40).^38^ Another prospective pregnancy cohort study conducted in the United Kingdom found that women who showed relatively higher GWG were more likely to have offspring with greater BMI, and greater pre-pregnancy weight was associated with greater offspring adiposity at the age of 9 years.^39^ Another multicenter, multiethnic cohort study suggested that the odds of overweight in the offspring at age 7 were 48% greater for children of mothers who gained excessive weight than for children of mothers who gained adequate weight (OR 1.48; 95% CI, 1.06–2.06).^40^ These and our findings emphasize the importance of appropriate weight gain during gestation in the prevention of childhood overweight and obesity. Moreover, the risk of childhood overweight was highest in pre-pregnancy overweight/obese mothers (OR 2.22; 95% CI, 1.79–2.76) in our study, highlighting the need to include weight counseling in preconception care.

Joint associations of maternal pre-pregnancy BMI and GWG with the risk of childhood overweight were also considered in our study, and we used children born to mothers with pre-pregnancy normal weight and also with adequate GWG as the reference. Children born to mothers with pre-pregnancy normal weight or overweight/obesity and also with excessive GWG had a higher risk of childhood overweight at 3–6 years old. Moreover, children born to mothers with pre-pregnancy overweight/obesity and also with adequate GWG also has a higher risk of childhood overweight at 3–6 years old. Our result can be seen as an extension of the relationship between GWG and infancy overweight risk.^41^

The pregnant women in our study had lower mean pre-pregnancy BMI (20.3 kg/m^2^) and lower mean GWG (11.7 kg) than American women (24.6 kg/m^2^ and 15.6 kg, respectively)^29^ and German women (22.6 kg/m^2^ and 14.3 kg, respectively).^42^ More than half (50.6%) of the women in our study gained inadequate weight during pregnancy and 14.5% excessive weight, while 14% of American women gained inadequate weight and 51% excessive weight,^29^ and 27.4% of German women gained inadequate weight and 37.0% excessive weight.^42^ In particular, we found that farmer mothers and mothers from the elementary or no education group were more vulnerable to inadequate weight gain during pregnancy. They were more likely to have imbalanced diets and poor knowledge of nutrition due to their relatively low-income status than women in other groups. The situation was opposite for mothers in Japan; Japanese women from higher socioeconomic backgrounds appeared to be at greater risk for inadequate weight gain.^43^ Slender women may be more sensitive to fashion trends, so they consciously try to keep fit even if they are pregnant.^43^ In addition, the children in our cohort had a much lower prevalence of overweight compared to children in Western societies, such as the United States and the European Union countries.^5^^,^^6^ These differences seem to support the notion that there is an association between maternal GWG and childhood overweight.

Across the age groups of children, the proportion of mothers in each GWG category and the proportion of children’s overweight look quite different. The proportion of mothers with excessive GWG and children’s overweight were higher in the younger child age group than among older groups. Aside from genetic and environmental factors, the cultural and historical belief that a fat child symbolizes prosperity of the family may have played an important role in this situation. As time passed, we speculated that this belief may have generated a trend in mothers gaining more weight during pregnancy and feeding their children more high-calorie food than mothers before. So it was not surprising that we observed this trend across child age groups.

We noticed that the higher pre-pregnancy BMI groups had higher proportions of mothers who gained excessive weight during pregnancy than lower-BMI groups. In the higher pre-pregnancy BMI groups, mothers may be more likely to eat higher calorie foods, do less physical activity, and use automobiles more because of life habits. In addition, these mothers may have failed to maintain their fitness before becoming pregnant. They may have continued these habits even after becoming pregnant, which could have resulted in gaining more weight during pregnancy than mothers from lower pre-pregnancy BMI groups. As maternal pregravid overweight/obesity and excessive weight gain during pregnancy are not perceived as a threat to the baby, mothers are unlikely to take measures to avoid these states. Relevant knowledge of prevention of these conditions is urgently needed among women of child-bearing age.

We also noticed that the risk of childhood overweight was highest among pre-pregnancy overweight/obese mothers. This may be due to the genetic association between parents and children. Indeed, reviews of the data on twin studies and on responses to chronic overfeeding and negative energy suggest that up to 70% of the susceptibility to gain weight may be inherited.^44^^,^^45^ However, children lived with their overweight/obese mothers and therefore shared a similar environment. The fact that the higher pre-pregnancy BMI groups had higher proportions of mothers who gained excessive weight during pregnancy may make the situation worse. However, the genetic predisposition towards overweight among children of overweight/obese mothers is not modifiable. Therefore, changes to diet and physical environment are essential if we want to have an effective impact.^46^^,^^47^ Other potential mechanisms of childhood obesity need to be explored to curb the epidemic.

It was also interesting that 54.9% mothers were primigravid, while 95.4% mothers were primiparous. The reason may be that unplanned pregnancies are common in China and the induced abortion rate is high due to the one-child policy. On the one hand, many families chose to abide by the one-child policy because the parents believed that they should “give birth to fewer children, but give them better care and education”, though the policy allows locally interpreted variations regarding having two or more children.^48^ On the other hand, the abortion rate of unmarried women in some cities has been found to be higher than that of married women.^49^ As a result, induced abortion is a common practice among women who seek to limit the number of children and to terminate unplanned pregnancies. In the whole nation, the ratio of the national abortion rate to normal birth rate is 0.6, according to the director of the Center for Clinical Research and Training at the Shanghai Institute of Planned Parenthood. Against this background, each household has fewer children, who have more chance to be overfed and become overweight in the long run.^50^

Because GWG is a modifiable factor that can be controlled when adequate prenatal nutritional counseling is provided, and because trimester-specific GWG can predict the likelihood of overweight of children, it should be possible to identify women at risk of inappropriate GWG as early as the second trimester.^51^ Monitoring weight gain throughout pregnancy, especially the early weeks of gestation, would help to improve the health outcomes of children, although the majority of health care counseling on GWG and nutrition is given to obese patients.^52^^–^^54^ A review by Guelinckx et al showed that only two out of seven intervention trials focusing on GWG, nutrition, and physical activity achieved a significant decrease in GWG.^55^ This means that we need to formulate effective strategies to help overweight and obese pregnant women live a healthy life and gain an appropriate amount of weight during pregnancy.

The current study was prospective in design, and the exposure variable was collected before outcomes were known. This helps to minimize the recall bias that is often present in retrospective studies, in which women may provide wrong values for weeks of gestation, child birth weight, and maternal weight and height due to deficiencies in memory. For example, in our study, birth weight was an important confounding factor. Because the follow-up date had been several years from the date of child birth, the mother could probably only remember the approximate value (eg, 3000 g) instead of an accurate value (eg, 2960 g). In addition, the present study included a large sample, which enabled us to conduct stratified analyses by maternal pre-pregnancy BMI, which strengthened the findings of our study. Furthermore, the cohort data were obtained in a standardized manner by health care professionals rather than self-reported. There are two limitations in our study that warrant mention. One limitation is that the pre-pregnancy BMI was calculated by the premarital weight and height. However, since about 86% women got pregnant within a year (mean [SD], 5.5[6.2] months) after the premarital assessment, we believe that the weight of mothers did not change significantly from the time of the premarital assessment to the time of conception, since the premarital assessment is conducted immediately before marriage in China. Another limitation was that information on breastfeeding, childhood nutrition, and physical activity was not collected in this study. Because our sample was mainly from counties or cities where lifestyle factors may not be significantly different, the impact, if any, is expected to be small. Future studies should consider the effects of these factors.

### Conclusion

Greater maternal GWG was associated with greater BMI of children at the age of 3–6 years, and the risk of childhood overweight was the highest for children of mothers who were overweight/obese before pregnancy and gained excessive weight during gestation. These findings indicate that childhood obesity prevention should be a component of preconceptional and prenatal care, which means that both the maternal pre-pregnancy BMI and GWG should be adjusted to appropriate values.
